# Effect of the environment on horizontal gene transfer between bacteria and archaea

**DOI:** 10.7717/peerj.3865

**Published:** 2017-09-29

**Authors:** Clara A. Fuchsman, Roy Eric Collins, Gabrielle Rocap, William J. Brazelton

**Affiliations:** 1 School of Oceanography, University of Washington, Seattle, WA, United States of America; 2 College of Fisheries and Ocean Sciences, University of Alaska—Fairbanks, Fairbanks, AK, United States of America; 3 Department of Biology, University of Utah, Salt Lake City, UT, United States of America

**Keywords:** Horizontal gene transfer, Archaea, Temperature, Oxygen utilization, Genome-size correction, Bacteria

## Abstract

**Background:**

Horizontal gene transfer, the transfer and incorporation of genetic material between different species of organisms, has an important but poorly quantified role in the adaptation of microbes to their environment. Previous work has shown that genome size and the number of horizontally transferred genes are strongly correlated. Here we consider how genome size confuses the quantification of horizontal gene transfer because the number of genes an organism accumulates over time depends on its evolutionary history and ecological context (e.g., the nutrient regime for which it is adapted).

**Results:**

We investigated horizontal gene transfer between archaea and bacteria by first counting reciprocal BLAST hits among 448 bacterial and 57 archaeal genomes to find shared genes. Then we used the DarkHorse algorithm, a probability-based, lineage-weighted method (Podell & Gaasterland, 2007), to identify potential horizontally transferred genes among these shared genes. By removing the effect of genome size in the bacteria, we have identified bacteria with unusually large numbers of shared genes with archaea for their genome size. Interestingly, archaea and bacteria that live in anaerobic and/or high temperature conditions are more likely to share unusually large numbers of genes. However, high salt was not found to significantly affect the numbers of shared genes. Numbers of shared (genome size-corrected, reciprocal BLAST hits) and transferred genes (identified by DarkHorse) were strongly correlated. Thus archaea and bacteria that live in anaerobic and/or high temperature conditions are more likely to share horizontally transferred genes. These horizontally transferred genes are over-represented by genes involved in energy conversion as well as the transport and metabolism of inorganic ions and amino acids.

**Conclusions:**

Anaerobic and thermophilic bacteria share unusually large numbers of genes with archaea. This is mainly due to horizontal gene transfer of genes from the archaea to the bacteria. ****In general, these transfers are from archaea that live in similar oxygen and temperature conditions as the bacteria that receive the genes. Potential hotspots of horizontal gene transfer between archaea and bacteria include hot springs, marine sediments, and oil wells. Cold spots for horizontal transfer included dilute, aerobic, mesophilic environments such as marine and freshwater surface waters.

## Introduction

Horizontal gene transfer (HGT) is an essential aspect of microbial evolution, but its mechanisms, rates, and consequences are poorly understood and difficult to quantify (Boto, 2009). Some organisms are more likely to engage in horizontal gene transfer than others. For HGT to occur, the transferred DNA has to be incorporated into the genome after uptake. For this reason, HGT is more common among closely related organisms (Kloesges et al., 2011). Intra-domain gene transfer seems to have been particularly important in bacterial evolution (Wolf et al., 2012). However, HGT does occur between archaea and bacteria and is more easily detected in such cases. Since the apparatus for transcription and translation are significantly different between archaea and bacteria, only transferred genes with strong benefits would be retained in their new genome, as their initial expression is likely to be low (Lawrence & Hendrickson, 2003). There is abundant evidence of HGT of key genes between bacteria and archaea adapted to live in similar environments: hot acid (Fütterer et al., 2004), acid mine drainage (Guo et al., 2015), extremely high salt (Mongodin et al., 2005), marine (Frigaard et al., 2006; Brochier-Armanet et al., 2011), high mercury (Barkay et al., 2010), and high temperature (Rhodes et al., 2011). Whether HGT is limited by geography has not been well investigated, but it likely spans oceans and continents. HGT between archaea and bacteria can be accomplished by acquiring free DNA from the environment, which implies greater proximity between the two parties, or by transfer of DNA from viruses or gene transfer agents. A comparison of viral metagenomes across the world’s oceans revealed that viruses are widely dispersed, though there is a relationship between geographic and genetic distance (Angly et al., 2006). Though most viruses are specific to either bacteria or archaea, *Caudovirales* viruses may be able to infect both bacteria and archaea (Prangishvili, Forterre & Garrett, 2006), and little is known about the range of gene transfer agents (McDaniel et al., 2010).

Multiple methods of detecting HGT have indicated that it can be influenced by the lifestyles of the organisms involved. One analysis of recent HGT, as determined by identification of identical 500 nucleotide blocks, indicated that ecology was more important than phylogeny; oxygen tolerance and pathogenicity were identified as the strongest factors (Smillie et al., 2011). Another study identified several hundred phylogenies of single genes (out of 7,000) that reflected the lifestyles of the organisms rather than their taxonomy (Schliep et al., 2011). For example, in 597 gene trees, hyperthermophiles and non-hyperthermophiles were better discriminated than were archaea and bacteria (Schliep et al., 2011). Rhodes et al. (2011) also detected increased transfer between thermophiles. However, when halophilic archaea were examined with the same technique, they appeared to have predominantly exchanged genes with non-halophilic microbes (Rhodes et al., 2011). It is possible this could be related to an ancient acquisition of 1,000 bacterial genes that caused Haloarchaea to differentiate from methanogens (Nelson-Sathi et al., 2012). Contrastingly, Gophna et al. (2015) found a negative correlation between growth temperature and gene transfer. In short, evidence indicates that horizontal gene transfer is affected by lifestyle differences, particularly growth temperature, oxygen utilization and pathogenicity.

Many similar studies, however, have reported that genome size is the most important factor determining the amount of horizontal gene transfer. In a study utilizing phylogenetic trees to examine HGT in eight genomes, genome size and %GC were the most significant factors determining the number of horizontally transferred genes, rather than more environmentally-relevant factors (Jain et al., 2003). Presence-absence patterns discordant with species trees have also indicated that large genomes have more horizontally transferred genes than small genomes (Cordero & Hogeweg, 2009; Gophna et al., 2015). A small genome is an adaptive strategy that allows a microbe to use fewer nutrients and resources. Consequently, microbes with smaller genomes expel unused genes (Giovannoni et al., 2005). For example, in a well studied group of archaea, *Methanosarcina mazei* has the smallest genome of the sequenced *Methanosarcina* and has clearly shed transferred genes found in other members of the genus (Garushyants, Kazanov & Gelfand, 2015). Gene loss is more common than gene gain over the evolution of both bacteria and archaea (Wolf et al., 2012). On the other hand, a large genome is an adaptive strategy in which excess genes are retained longer (Chang et al., 2011) in order to allow greater versatility (Guieysse & Wuertz, 2012). Thus, counting the absolute number of transferred genes present in a genome does not necessarily reflect the rate of horizontal transfer into that organism. This, however, could happen at a much larger scale when comparing organisms from very different environments. In order to compare bacteria from a range of living conditions, we look for bacteria with unusual levels of HGT with archaea than predicted by their genome size.

## Methods

A total of 448 bacterial and 57 archaeal genomes (Table S1) were compared using reciprocal BLAST hits (BLAST version 2), where each BLASTp hit had to have an e-value of less than or equal to 1e−10 and the alignment length was >50% of the query (Table S2). Genomes were downloaded from the NCBI ftp site in 2009 and in cases where a microbe had one or more plasmids, those plasmids were included with the genome. All genomes were 100% complete and closed and no two genomes are from the same species.

Information about each organism and its temperature, oxygen, and salt preferences were first found on the IMG website (http://img.jgi.doe.gov). If information was missing, we next looked at the JGI genome portal summary for the organism. Finally, information was taken from the primary literature. The salt preferences of many non-halophilic or moderately halophilic bacteria were inferred from the environmental conditions from where they were isolated, where the marine environment was considered moderately halophilic (Table S1).

### Removing the genome size effect

A nonlinear genome size effect has been observed for pairwise comparisons of shared genes between bacteria and archaea (Snel, Bork & Huynen, 1999; Fuchsman & Rocap, 2006). The genome size of the bacteria is a much more important factor than the genome size of the archaea in this nonlinear genome size effect (Fuchsman & Rocap, 2006). To account for variation in reciprocal best hits (RBHs) due to genome size of bacteria, a log–logistic function function was fit to the data. Log–logisitic model selection was conducted using zunzun (http://www.github.com/zunzun/zunzunsite), guided by the Akaike information criterion. The four-parameter log–logistic function was chosen because it performed well in fitting both the long tail of large bacterial genomes and the sigmoidal rise observed among small bacterial genomes. A four-parameter log–logistic function of the form

\begin{eqnarray*}f(x)=c+ \frac{d-c}{1+1{0}^{b\ast (\log \nolimits (x)-\log \nolimits (e))}} \end{eqnarray*}

was chosen and was fitted for each archaeon, with bacterial genome size as the predictor and RBHs as the response variable. The parameters b, c, d, and e were estimated for each archaea in R using the non-linear fitting function ‘drm‘ in the ‘drc‘ package. The fit of the log–logistic function for all archaea individually can be seen in Fig. S1. The R code and RBH dataset necessary to reproduce the regression modeling and visualization is freely available at http://www.github.com/rec3141/hgtbaab.

In addition, we examined the Korbel normalization, which includes the genome size of both bacteria and archaea:

\begin{eqnarray*}\text{weighted average}= \frac{\sqrt{2}\times A\times B}{{A}^{2}+{B}^{2}} \end{eqnarray*}

where A and B are the genome size of the archaeal and bacterial genomes (Korbel et al., 2002). The Korbel fit was similar to our log–logistic function, but was found to be less accurate for bacteria with >5,000 genes (Fig. S2). Therefore, we used the log–logistic function in all our analyses.

The residual numbers of RBHs in each genome were calculated by subtraction of the predicted fit line, which we’ll refer to as ‘residual’ RBHs. The effect of genome size was successfully removed by this subtraction, and number of genes and residuals were no longer correlated (Table S3). Residuals were not normally distributed due to the non-random contributions of horizontally transferred genes (Fig. S3). For each archaeon, bacteria with residual RBH counts greater than 1.96 standard deviations from 0 were defined as *significantly enriched* in shared genes and considered to form an *enriched pair* with that archaeon (Table S3).

Since this residual calculation was done for each of 57 archaea, it is susceptible to multiple testing issues. From the Bernoulli process, there is a 14% chance that one out of 441 bacteria would be erroneously considered enriched with one out of 57 archaea. However, one would expect zero bacteria to randomly be enriched in comparisons with three or more archaea. In our dataset, only four bacteria were enriched in <3 archaea and all four were also supported by enrichment in horizontally transferred genes (below). Therefore, we believe our confidence interval was conservative enough to avoid false positives.

Bacteria-bacteria dissimilarity matrices were constructed with residual RBHs using the Bray–Curtis algorithm. These matrices were used in ANOSIM analyses (Clarke, 1993) with the Primer 6 program (http://www.primer-e.com) to examine the significance oxygen utilization and temperature. There was no significant difference in bacterial genome size between growth temperature groups (mesophiles: 3,857  ± 1,753 genes; thermophiles: 2,491  ± 727 genes) or between oxygen utilization groups (anaerobes: 2,810  ± 1,039 genes; aerobes 4,439  ± 1,811 genes). Extremely halophilic bacteria had an average genome size of 2,806  ± 331 genes.

To examine the significance of %GC along with oxygen utilization and temperature, primary residual RBHs were calculated and re-fitted by multiple linear regression with ‘lm‘ using %GC content and bacterial genome size as predictors (Fig. S4). Secondary residual RBHs were then calculated for use in statistical analyses where noted.

### Mantel test

All 16S rRNA sequences were obtained from the GreenGenes database (DeSantis et al., 2006). Sequences were aligned, and distances were calculated with mothur (Schloss et al., 2009). The purpose of the Mantel test was to test whether deeply-branching bacteria share more genes with archaea than more divergent bacteria. Therefore, for the Mantel test, only the distances between bacteria and the archaeal root were included in order to remove the phylogenetic signal from bacteria-bacteria relationships. The phylogenetic distances between each bacteria and the archaeal root were used to create a bacteria-bacteria Euclidean distance matrix with Primer. The Mantel test compared a bacteria-bacteria matrix of phylogenetic distance to archaea with a bacteria-bacteria matrix of shared genes with archaea. Mantel tests were performed with the R package “vegan”.

### DarkHorse algorithm

DarkHorse is a statistical method to look for horizontal gene transfer, using lineage probability index ranking scores (LPI), which inversely reflect phylogenetic distance between the query amino acid sequence and its closest database match (Podell & Gaasterland, 2007; Podell, Gaasterland & Allen, 2008). The lineage of each gene is compared to the lineage of the entire organism. The genes flagged by the DarkHorse algorithm have not been checked with phylogenetic trees, so they should be considered potential horizontally transferred genes. However, the phylogenetic distance between bacteria and archaea is very large, so we are examining genes with very low LPI numbers (below 0.2 (Podell & Gaasterland, 2007)), which increases the robustness of our analysis. Scores at the lowest end of the LPI range are the most likely to be real horizontally transferred genes because their best hit is quite distant from the query organism (Podell & Gaasterland, 2007).

Files including LPI numbers for all available proteins in each of the archaeal and the non-pathogenic bacterial genomes were downloaded from the DarkHorse database at http://darkhorse.ucsd.edu at the genus level comparison (Podell, Gaasterland & Allen, 2008). Using a genus level comparison to find transferred genes means that genes shared at a family or class level would not be considered horizontally transferred. This setting may bias our results away from ancient transfer events. For example, the transfer of bacterial genes that allowed the class Haloarchaea to form from methanogens (Nelson-Sathi et al., 2012) would not be marked as transferred in our scheme.

Proteins in reciprocal best hit files were matched to LPI numbers from DarkHorse for both the bacteria and archaea. If the LPI number was below 0.2 for either the bacteria or the archaea, the reciprocal best hit was tagged as horizontally transferred (Table S4). For each archaeon, each bacterium with a horizontally transferred gene count more than 1.96 standard deviations from the mean was defined as *significantly enriched* in horizontally transferred genes and considered to form an *enriched pair* with that archaeon. This technique is susceptible to multiple testing issues, but enrichment with three or more archaea is considered robust. Three bacteria were >1.96 standard deviations from the mean for only one archaea and were not substantiated by enrichment in shared genes (above). These three bacteria were removed from further analysis as potential false positives.

### COGs

Files linking proteins to COGs or Clusters of Orthologous Genes were downloaded from NCBI ftp site. The COG collection used was originally created in 2003 (Tatusov et al., 2003) and then used to identify COGs in subsequently sequenced genomes. The numbers of RBH in each category can be seen in Table S5. Fifteen categories had enough shared genes in them to examine statistically. COG categories were examined both with and without removal of the genome size effect. Before ANOSIM analyses, a four-parameter log–logistic function was fit between bacteria and each archaea for each COG category to remove the effect of genome size, as described above. The removal of the correlation of RBH and genome size was checked for each COG and archaea and was successful in all cases (*R*^2^ < 0.001). Additionally, to further take GC into account, the effect of genome size and GC were simultaneously removed from the residuals for each archaea by multiple linear regression using the number of genes in the bacterial genome, reciprocal BLAST hits, and GC content. ANOSIM (Clarke, 1993) analyses were done with the Primer 6 program (http://www.primer-e.com).

## Results and Discussion

**Figure 1:**
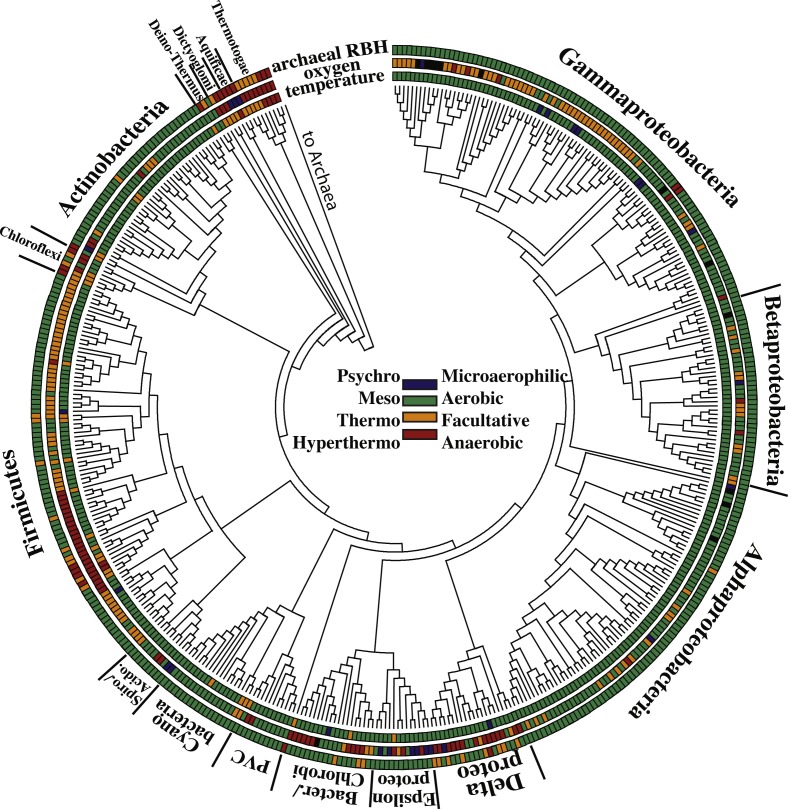
Tree of all the bacteria used in this study. For names see Table S1. The inner ring is the growth temperature range of the bacteria. The middle ring is the oxygen utilization potential of the bacteria. The outer ring represents the frequency of archaea-bacteria pairs enriched in reciprocal best hits (RBHs), where green represents 0–3 pairs, orange 4–24 pairs, and red 25+ pairs.

**Figure 2:**
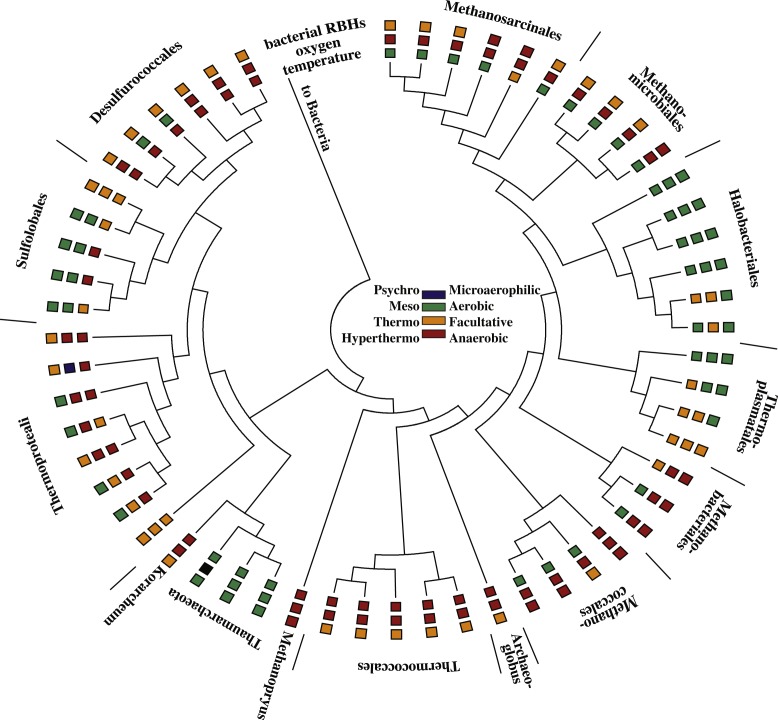
Tree of all the archaea used in this study. For archaeal names see Table S1. The inner ring is the growth temperature range of the archaea. The middle ring is the oxygen utilization potential of the archaea. The outer ring represents the frequency of archaea-bacteria pairs enriched in reciprocal best hits (RBHs), where green represents 0–18 pairs, orange 19–25 pairs, and red 25–31 pairs.

Of the 448 bacterial genomes used in this study, 273 are from non-pathogens, 81 were anaerobes, 42 were thermophilic or hyperthermophilic (optimum growth temperature >50 °C; abbreviated as “(hyper)thermophilic”) (Fig. 1; Table S1). Regarding salinity, there were 72 moderately halophilic bacteria and 6 extremely halophilic bacteria (Table S1). Most of the bacteria in this dataset are aerobic mesophiles, even when pathogens are not included (Table S1). Of the archaea, 34 were anaerobes and 34 were (hyper)thermophilic (Fig. 2: Table S1). Twenty-three archaea were moderately halophilic while six were extremely halophilic (Table S1). Only four archaea were mesophilic aerobes living at normal salt conditions (Fig. 2: Table S1). However, this reflects current knowledge of archaeal diversity, i.e., few major taxonomic divisions of archaea include representatives from aerobic non-extreme environments with complete genome sequences. For example, there are only two main types of archaea commonly found in the oxic ocean: Thaumarcheota and Marine Group II Euryarcheota (Karner, DeLong & Karl, 2001). Marine Group III Euryarcheota and Marine Group IV Euryarcheota are also occasionally found in the ocean but have not been sequenced (Zhang et al., 2015). Thaumarcheota are the main type of archaea in oxic soils (Bates et al., 2011) and in oxic freshwater (Reitschuler, Hofmann & Illmer, 2016). There are at present only 11 known genera in the phylum Thaumarcheota, four of which are represented in our dataset (one is thermophilic). There are presently two known types of Marine Group II Euryarcheota (Zhang et al., 2015), one of which is included in our dataset.

**Figure 3:**
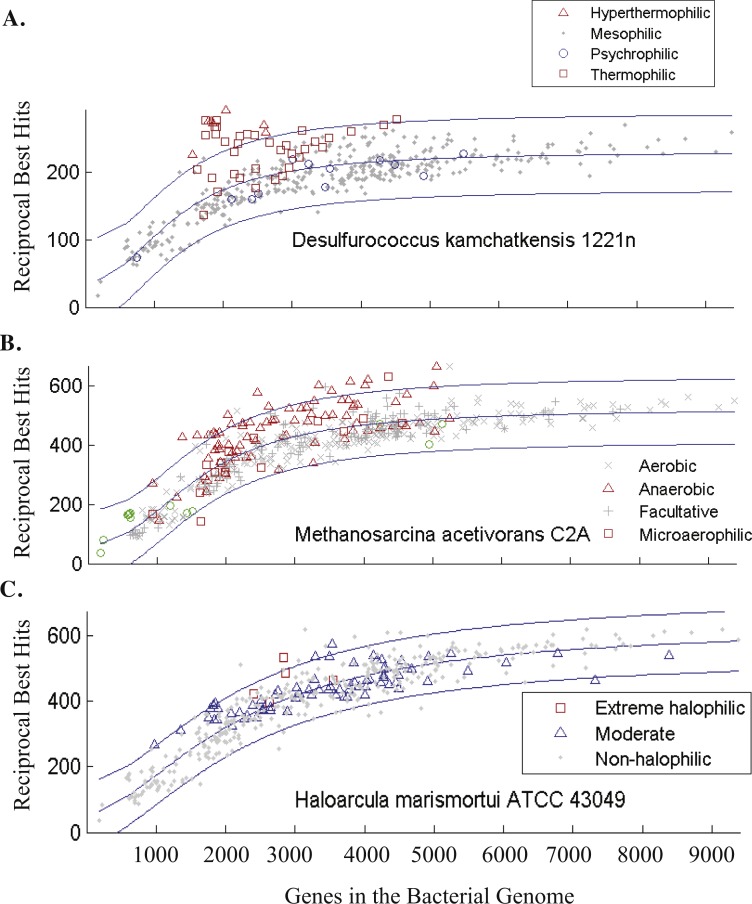
Genome size and number of reciprocal best hits fit using non-linear regression. Examples of (A) hyperthermophilic, (B) anaerobic, and (C) extremely halophilic archaea. The number of shared genes between archaea and bacteria were fitted using non-linear regression (black lines) and 1.96 standard deviations from than the mean were calculated for the residuals (grey lines) to visualize the range. Bacteria-archaea pairs with reciprocal best hit (RBH) counts more than 1.96 standard deviations greater than the mean are defined as *enriched* in RBHs. Green marker indicate bacterial endosymbionts where their oxygen utilization was unclear. The X axis is identical in all three graphs and labels are found in part (C).

### Eliminating the genome size effect

Genome size can confound the quantification of HGT because the number of genes an organism accumulates is an adaptive strategy influenced by many other factors such as nutrient regime. A small genome allows a microbe to use fewer nutrients and to have a favorable surface to volume ratio, so microbes adapted to smaller genomes expel unused genes (Giovannoni et al., 2005). A large genome in which excess genes are retained longer allows a microbe greater versatility (Chang et al., 2011; Guieysse & Wuertz, 2012). Merely counting numbers of transferred genes does not take into account these lifestyle differences. In this paper we endeavored to determine which bacteria have engaged in more HGT with archaea than predicted by their genome size. A nonlinear genome size effect has been observed for pairwise comparisons of shared genes between bacteria and archaea (Snel, Bork & Huynen, 1999; Fuchsman & Rocap, 2006). The genome size of the bacteria is a much more important factor than the genome size of the archaea in the nonlinear genome size effect (Fuchsman & Rocap, 2006). After determining shared genes from reciprocal best BLAST hits (Table S2), we explored two ways to predict the expected number of shared genes for each bacterial genome size: (1) the Korbel weighted average that utilizes both archaeal and bacterial genome size and (2) an empirical log–logistic function. While the fits were similar for bacterial genome sizes <5,000 bp, the empirical log–logistic function was superior for genomes >5,000 bp (Fig. S2) so was used for further calculations (Fig. 3, Fig. S1). Here, we denote archaea-bacteria pairs that have significantly more (>2 standard deviations from the mean calculated with the log–logistic function) reciprocal best hits (RBHs) than expected based on genome size to be *enriched* in shared genes (Table S3). This technique is particularly useful for identifying unusually large numbers of shared genes in bacteria with small genomes.

### Identifying horizontal gene transfer

Genes can be shared between archaea and bacteria either due to horizontal gene transfer or due to vertical inheritance from their common ancestor. To test for the extent to which vertical inheritance can explain the numbers of shared genes between archaea and bacteria, we used a Mantel test to compare a similarity matrix derived from each bacterial genome’s number of shared genes with each archaeal genome to a similarity matrix derived from each bacterial genome’s 16S rRNA genetic distance to archaeal 16S rRNA. The correlation between the two matrices is significant (*R* = 0.176  *p* < 0.0001), but the *R* value is not high. This result implies that some, but not all, of the shared genes between archaea and bacteria were inherited from the last common ancestor, which is not surprising.

To disentangle vertical inheritance and horizontal transfer, we used the DarkHorse algorithm, which searches for horizontally transferred genes using a probability-based, lineage-weighted method in which the lineage of each gene is compared to the lineage of the entire genome (Podell & Gaasterland, 2007). The phylogenetic distance between bacteria and archaea is very large, so we are only examining genes with extremely large lineage differences from the entire genome, which increases the robustness of our results. By cross-referencing our reciprocal best hit data with the DarkHorse results, we can flag some genes as potentially horizontally transferred (Table S4).

**Figure 4:**
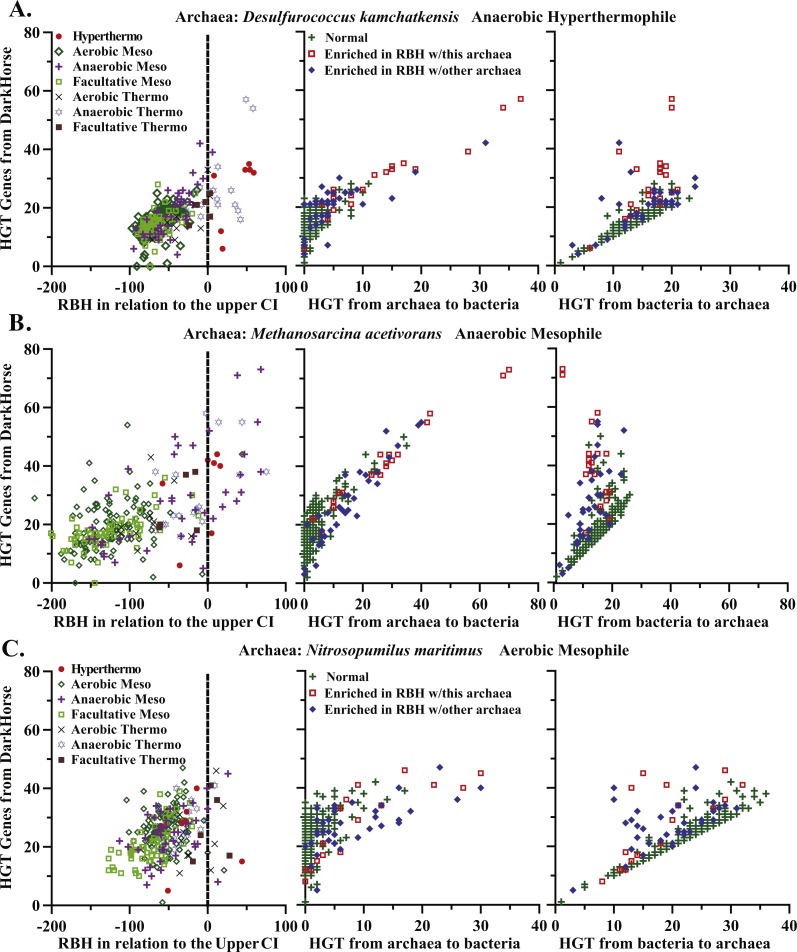
Comparison of shared and transferred genes. Examples of HGT for anaerobic hyperthermophile *Desulforococcus kamchatkaensis* (A), anaerobic mesophile *Methanosarcina acetivorans* (B), aerobic mesophile *Nitrosopumilus maritimus* (C). In the first column the number of potentially horizontally transferred genes as determined from the DarkHorse algorithm is compared to the number of reciprocal best hits between bacteria and each archaeon. A positive value on the *x*-axis indicates the RBH count was greater than 1.96 standard deviations from the mean. In the second column, the number of potentially transferred genes is compared to the number of those genes transferred from archaea to bacteria. In the third column, the number of potentially transferred genes is compared to the number of those genes transferred from bacteria to archaea. Color-coding describes whether the bacterium forms a pair with the archaeon listed (red), with any archaea in this study (blue), or not with any archaea (green).

In general, the numbers of potentially horizontally transferred genes identified by DarkHorse are greater in bacteria that form archaea-bacteria pairs enriched in shared genes (Fig. 4 and Fig. S5). Over half (55%) of the bacteria in pairs with enriched RBHs also had greater than two standard deviations above the mean potential horizontally transferred genes identified by DarkHorse.

### Correlations with growth conditions

**Figure 5:**
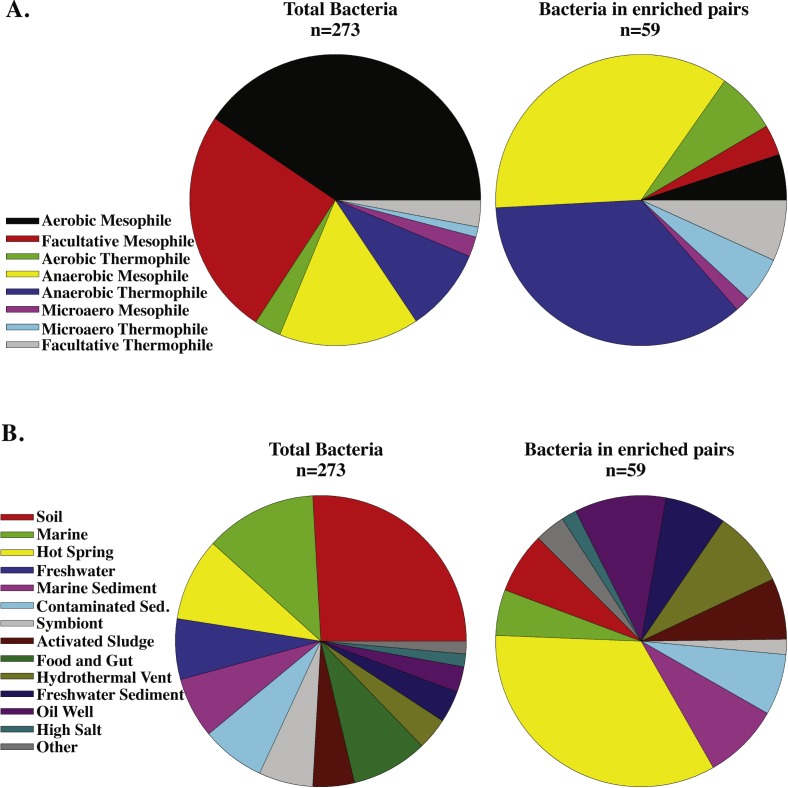
Comparison of bacteria enriched in shared genes with archaea to the composition of the database. Pie charts comparing the bacterial database, excluding pathogens, to the bacteria that formed pairs enriched in shared genes (RBHs) with at least one archaeon. (A) Color coded with oxygen utilization and growth temperature of the bacteria (B) Color coded with the environment where the bacteria was isolated.

After the effect of genome size is removed, interesting trends emerge between the number of shared genes and the lifestyles of archaea and bacteria. For example, the enriched archaeal-bacterial pairs involving *Desulfurococcus kamchatkensis*, an anaerobic hyperthermophilic archaeon, predominately include (hyper)thermophilic bacteria (Fig. 3A). Similarly, *Methanosarcina acetivorans*, an anaerobic mesophilic archaeon, primarily forms enriched archaeal-bacterial pairs with anaerobic bacteria (Fig. 3B). Contrastingly, the number of archaea-bacteria pairs involving extremely halophilic bacteria and *Haloarcula marismortui*, an extremely halophilic aerobic mesophilic archaeon, are not significantly more than expected for their genome sizes (Fig. 3C). In general, the bacteria that share unusually large numbers of genes with archaea (i.e., after correction for genome size) are predominantly anaerobic and/or thermophilic (Fig. 5A). Bacteria isolated from hot springs, oil wells, sediments and activated sludge were over-represented in this set of bacteria (Fig. 5B). This result is remarkable considering that most of the bacterial genomes used in this study were from aerobic mesophiles (Fig. 5A). These trends are also true in the converse; i.e., anaerobic and (hyper)thermophilic archaea are more likely to have large numbers of RBHs with bacteria (Fig. S6). In contrast, aerobic mesophilic archaea have a small number of RBHs with bacteria and form the fewest archaea-bacteria pairs enriched in RBHs (Fig. S6).

**Figure 6:**
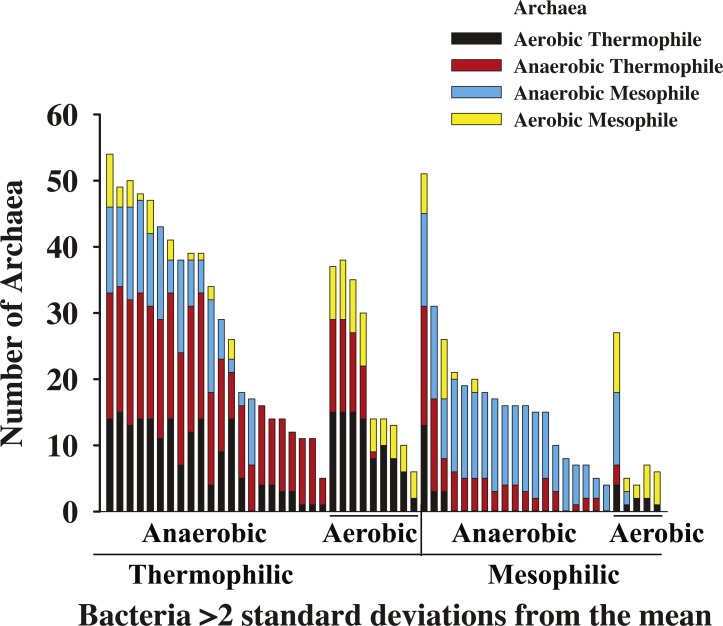
Phenotypes of bacteria and archaea that are enriched in shared genes. The bacteria that formed pairs enriched in shared genes (RBHs) with three or more archaea and the phenotypes associated with those archaea. Labels on the *x* axis describe the bacteria while color coding describes the archaea.

To investigate more precisely which types of archaea and bacteria are more likely to share genes, we examined whether the archaea and bacteria in each enriched pair shared their growth conditions and habitats (Fig. 6). Indeed, in general, anaerobic bacteria share unusually large numbers of genes with anaerobic archaea, and (hyper)thermophilic bacteria share unusually large numbers of genes specifically with (hyper)thermophilic archaea (Fig. 6). One explanation of this result is that bacteria and archaea that live in similar environmental conditions are more likely to share genes with each other. However, this does not seem to apply to aerobic mesophilic bacteria. While all hyperthermophilic bacteria, 80% of thermophilic bacteria were enriched with at least one archaeal partner, and 56% of anaerobic bacteria were enriched in shared genes, Very few aerobic mesophilic bacteria were ever included in enriched pairs, even with aerobic mesophilic archaea (Fig. 6), though it is true that we only have 4 aerobic mesophilic archaea in our dataset. Pathogens, either anaerobic or aerobic, were never enriched in RBHs. To validate these findings statistically, a series of ANOSIM tests were conducted to investigate factors associated with the number of shared genes between bacteria and archaea (Table 1). Since many of the bacteria and archaea are both anaerobic and thermophilic, we used a two-way ANOSIM to take into account both oxygen and temperature simultaneously. Indeed, each factor independently had a significant correlation with the number of shared genes between archaea and bacteria (*p* = 0.001; Table 1).

**Table 1:** Statistical analyses. Overall summary of ANOSIM results. The groups for GC content were: low GC = 30–45, medium = 46–55, high = 56–70.

ANOSIM	*R*	*P* value
**Temperature (1 way)**	0.614	0.001
**Oxygen (1 way)**	0.213	0.001
**GC content (1 way)**	0.082	0.001
**Salt (1 way)**		Not significant
**pH**	0.249	0.008
**Metabolism (1 way)**	0.045	0.004
Pathogen vs Autotroph	0.224	0.001
Pathogen vs Heterotroph	0.023	0.006
Heterotroph vs Autotroph		Not significant
**Oxygen (2 way w/temp)**	0.170	0.001
Anaerobic vs Aerobic	0.405	0.001
Anaerobic vs facultative	0.324	0.001
Aerobic vs facultative		Not significant
**Temperature (2 way w/O**_**2**_**)**	0.547	0.001
Hyperthermo vs Meso	0.654	0.001
Thermo vs Meso	0.517	0.001
Hyperthermo vs Thermo		Not significant
**Oxygen (2 way w/GC)**	0.227	0.001
**GC content (2 way w/O**_**2**_**)**	0.099	0.001
Low vs High	0.235	0.001
Medium vs High	0.088	0.001
Low vs Medium		Not significant
**pH (2 way w/Temp)**		Not significant
**Mantel Test with 16S Matrix**	0.176	0.000

Like the number of reciprocal best hits, the number of potentially transferred genes is highest among anaerobic or thermophilic bacteria and anaerobic or thermophilic archaea (Fig. 4 and Fig. S5). For Dehalococcoides sp. BAV1, 12–31% of total shared genes were horizontally transferred, and this was fairly typical for bacteria enriched in shared genes. The numbers of transferred genes were larger than the residual from the upper confidence interval for shared genes, but these two numbers are not necessarily comparable. However, it does seem likely that a large proportion of the extra shared genes in enriched archaea-bacteria pairs (i.e., those identified by our correction for genome size) were horizontally transferred.

**Figure 7:**
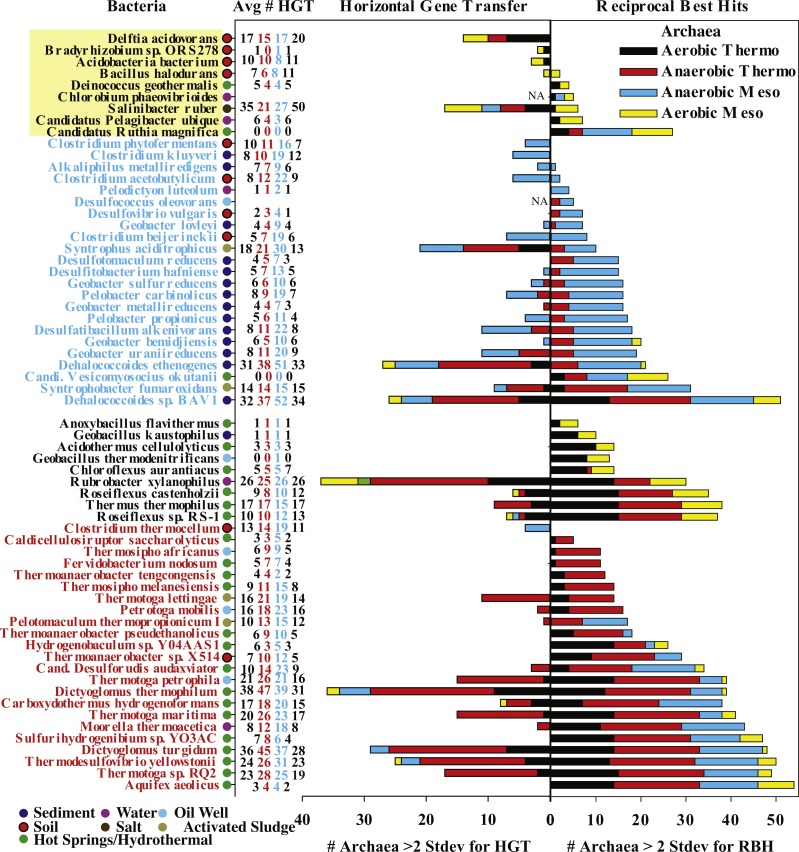
Examination of horizontal gene transfer in individual bacteria. Bacteria that form pairs with >3 archaea for either the number of residual reciprocal best hits (RBH) or potentially horizontally transferred genes, as determined by DarkHorse. The color coded bar graphs represent the number of archaea with which that bacterium forms enriched pairs (similar to Fig. 6). Bacterial names (left) are also color coded by bacterial oxygen utilization and growth temperature as in legend. Circles next to bacterial names indicate bacterial isolation environment. The column of numbers indicates the average number of horizontally transferred genes from archaea to bacteria for each category of archaea in the order aerobic thermophilic, anaerobic thermophilic, anaerobic mesophilic, and aerobic mesophilic archaea. The average number of HGT genes from archaea to bacteria for the entire dataset is four.

The DarkHorse results on horizontal gene transfer can be evaluated to determine the direction of transfer (i.e., from bacteria to archaea or from archaea to bacteria). Most documented cases of HGT between bacteria and archaea are predominantly unidirectional from bacteria to archaea (Wiezer & Merkl, 2005; Kanhere & Vingron, 2009; Nelson-Sathi et al., 2015). The number of transferred genes from bacteria to archaea may be five-fold larger than the number of genes transferred from archaea to bacteria (Nelson-Sathi et al., 2015). Our results agree with this trend for “normal” archaea-bacteria pairs (i.e., those that are not enriched in shared genes); these pairs generally have more genes transferred from bacteria to archaea (Fig. 4 column 3; Table S4). However, of the 58 bacterial genomes that form pairs for enriched numbers of shared genes, over half had 11 or more genes horizontally transferred from archaea to bacteria (Fig. 7), whereas the mean number of archaea to bacteria transfers for all genomes is only 4 ± 7. Anaerobic and (hyper)thermophilic bacteria have more transferred genes from anaerobic and (hyper)thermophilic archaea, respectively, than from aerobic or mesophilic archaea (Fig. 7). Some bacteria, such as *Dictyoglomus* and *Dehalococcoides*, have 30–50 horizontally transferred genes from archaea (Fig. 7). These results suggest that the unusually large numbers of shared genes in our enriched archaea-bacteria pairs is primarily due to an unusually large number of gene transfers from archaea to bacteria in anaerobic and (hyper)thermophilic conditions.

### Examination of halophily

Given that the numbers of shared genes are strongly associated with bacterial growth temperature and oxygen utilization, the lack of any significant correlation with halophily is surprising (Table 1) though consistent with results from Rhodes et al. (2011). There appears to be no difference in the numbers of shared genes between bacteria and archaea for non-halophilic and moderately halophilic bacteria. Of the six extremely halophilic bacteria, only two, *Salinobacter ruber* and *Chromohalobacter salexigens*, were ever enriched in RBHs. *Salinibacter ruber*, which is known to share genes with haloarchaea, including rhodopsins and a hypersalinity island (Mongodin et al., 2005), was enriched in shared genes with each of the six extremely halophilic archaea. *Salinibacter* also possessed many horizontally transferred genes from archaea (Fig. 7). *Salinibacter* was isolated from a salt cistern, which is the saltiest habitat known to harbor bacteria (Mongodin et al., 2005). None of the other extremely halophilic bacteria in this dataset reach the ultra-extreme halophily of *Salinobacter* (Hirschler-Rea, 2003; Hou et al., 2004; Mongodin et al., 2005; Auman et al., 2006; Hoeft et al., 2007). Thus *Salinobacter* is an exception among the six halophilic bacteria examined here in its extensive sharing and transferring of genes with archaea, and this may be related to its extreme halophily.

We do not deny that halophilic archaea have been changed by horizontal gene transfer with bacteria. However, the ancient transfer of 1,000 bacterial genes that allowed the class Haloarchaea to form from methanogens (Nelson-Sathi et al., 2012) would not be marked as transferred in our scheme because the genes are present in several genera. Additionally these genes don’t appear to be shared specifically with other modern sequenced halophilic bacteria.

### Correlation with genome %GC

Along with oxygen utilization and growth temperature range, bacterial genome %GC content was also a significant factor correlated with the number of shared genes (Table 1). As has been noted previously (Naya et al., 2002; Wu et al., 2012), in our dataset aerobic bacterial genomes have a significantly higher %GC (59%) than anaerobic bacterial genomes (45%; *p* < 0.001) but GC content does not correlate with oxygen utilization in archaea. Similar to Hurst & Merchant (2001), GC content does not correlate between temperature groups in either archaea or bacteria in our dataset. To verify that %GC is not confounding the effect described above for anaerobes and (hyper)thermophiles, we repeated our analyses after correcting for both genome size and genome %GC by multiple linear regression (Fig. S4). The magnitude of the %GC correction (average of 2.6 per 10% GC change) was much smaller than that for the original genome size correction (average 45 per 1,000 genes). Interestingly, a relationship between GC content and reciprocal blast hits was only significant for aerobic archaea (*p* < 0.01). A two-way ANOSIM of the secondary residuals indicated that oxygen utilization and growth temperature of the bacteria were still significant factors affecting numbers of RBHs (aerobic vs. anaerobic *R* = 0.382  *p* = 0.001; mesophilic vs. hyperthermophilic *R* = 0.676*p* = 0.001).

### Environment

To address the question of whether bacteria and archaea from the same habitats are more likely to engage in HGT, we categorized the DarkHorse and RBH results by the environment from which the archaea and bacteria were isolated.

**Figure 8:**
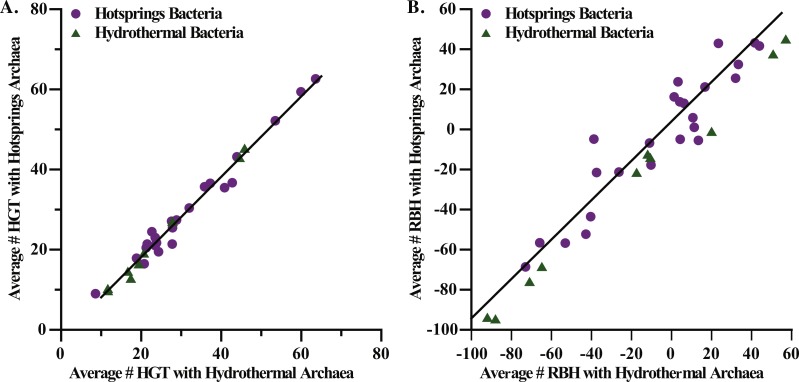
Comparison of transferred and shared genes with habitat. Comparison of transferred (A) and shared (B) genes with habitat. Particularly, we compare genes in hotsprings and hydrothermal vent bacteria to hotsprings and hydrothermal vent archaea. HGT stands for horizontally transferred genes, and RBH stands for reciprocal best hits or shared genes.

Habitats with similar temperature and oxygen characteristics, such as terrestrial hot springs and marine hydrothermal vents, have similar numbers of horizontally transferred genes and RBHs between bacteria and archaea (Fig. 8). A similar correspondence was observed with animal guts and sediments, both of which harbor a high proportion of anaerobic organisms involved in the degradation of complex organic matter (Fig. S7). These findings suggest that metabolic and physiological similarities could be more important in determining successful gene transfers between archaea and bacteria than co-habitation. However, our database does not include bacteria and archaea isolated from exactly the same area, which would be needed to examine this question more thoroughly.

Our findings do suggest that HGT is more common in some habitats than others. Oil wells, hot springs, and sediments are environments where bacteria and archaea share unusually large numbers of genes (Fig. 5B). 30% of bacteria with HGT genes >2 standard deviations above the mean were from hot springs, 32% were from sediments and 20% were from soil (Fig. 7). Thermophiles employ various mechanisms to mitigate DNA degradation at high temperatures, so thermophile-derived DNA may be more likely to persist both inside and outside of cells. This biochemical characteristic of thermophiles has been previously suggested as a possible cause for the greater prevalence of HGT events between thermophiles compared to halophiles (Rhodes et al., 2011). Furthermore, oil wells and hot springs are typically dominated by biofilm communities (Blenkinsopp & Costerton, 1991; Meyer-Dombard et al., 2011; Marks et al., 2012), which are known to facilitate HGT due to the high density and close physical proximity of metabolically active cells (Molin & Tolker-Nielsen, 2003; Aminov, 2011). Sediments and soil also have a high density of microbial cells (Christensen, Hansen & Sørensen, 1999; Schippers et al., 2012). Additionally, viral abundance and production in sediments is high compared to the water column (Danovaro et al., 2008), and more than half of the DNA in sediments is extracellular (Dell’Anno & Danovaro, 2005). Therefore, all of these factors (cell density, viral abundance, and extracellular DNA) may promote HGT in sediments.

### Functional characterization of shared and transferred genes

**Figure 9:**
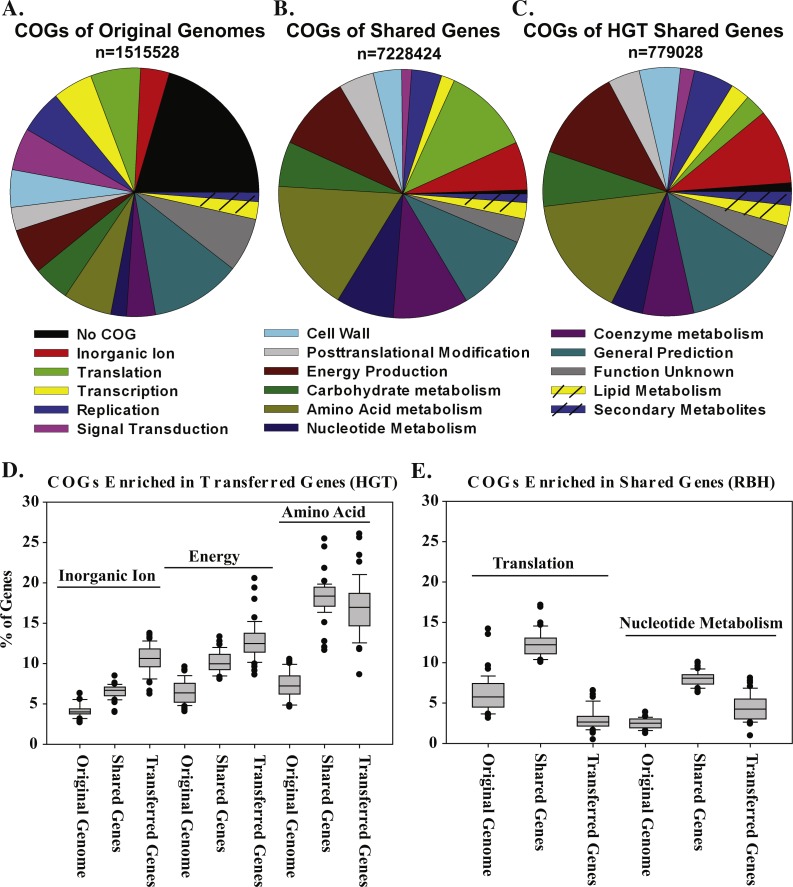
COG categories of genes shared between archaea and bacteria. (A) COG categories of all genes in all genomes, (B) all genes shared between bacteria and archaea as measured by RBHs, and (C) all genes identified as HGTs by DarkHorse. (D) Boxplot comparing COG cateogories that are enriched in HGTs. (E) Boxplot comparing COGs that are enriched in RBHs. In the boxplots, the lines represent the mean, points represent outliers, the edges of the box are the 25th and 75th percentiles and the whiskers represent the 90th percentile. Values in the boxplots are normalized by the appropriate total numbers of genes.

To gain more insight into the types of genes that may be shared more frequently between anaerobic and (hyper)thermophilic organisms, we examined the shared genes (RBHs) by functional category using the COG database (Tatusov et al., 2003) (Table S5). Some COG categories are more likely than others to contain genes shared between bacteria and archaea. Specifically, the categories Energy Production, Translation, Nucleotide Metabolism, and Coenzyme Metabolism contain a higher proportion of archaea-bacteria RBHs than that found in the total set of genes in all genomes (Fig. 9). Curiously, HGTs identified by DarkHorse are not enriched in these same categories; instead, they are more abundant in Inorganic Ion Transport and Metabolism (such as metal or sodium transporters), Energy Production and Conversion, and Amino Acid Transport and Metabolism (Fig. 9B). Consistent with previous findings that transferred transporters were enriched in *Methanosarcina* genomes (Garushyants, Kazanov & Gelfand, 2015), transporters were especially enriched in HGTs in our dataset: 15% of HGTs involved transporters of some kind compared to 5% in the whole dataset of all genomes. Indeed, transporters can be expected to confer immediate benefits to a cell after horizontal transfer.

Interestingly, archaea and bacteria share many genes in the Translation category, but these genes are not horizontally transferred (Fig. 9C). This is consistent with translation proteins being highly conserved between archaea and bacteria (Koonin et al., 2012) and is likely an example of genes shared from vertical descent. Nucleotide Transport and Metabolism genes are also frequently shared by archaea and bacteria but not horizontally transferred (Fig. 9C). Notably, Transcription and Signal Transduction genes do not seem to be shared between bacteria and archaea (Fig. 9). The lack of shared genes in these categories is not surprising (Cordero & Hogeweg, 2009), considering that archaea and bacteria employ completely different transcription machinery (Jun et al., 2011) and signal transduction pathways (Ulrich, Koonin & Zhulin, 2005).

The abundance of shared genes in each COG category was normalized for genome size using the empirical fit log–logistic function (Table S6). As with the total dataset, temperature, oxygen utilization and GC content were all significantly correlated with the number of shared genes in many COG categories (Table 2). However, the three variables were significant in determining the number of shared genes for different COG categories. %GC was only significant for COG categories Amino Acid Synthesis, Energy Production, and Nucleotide Synthesis. Interestingly, oxygen utilization was significant for more COG categories than temperature, including Transcription, Energy Production, and Coenzymemetabolism. Additionally the category Carbohydrate Metabolism was significant for temperature, but not oxygen utilization. These results demonstrate that the genes shared among anaerobes are different than the genes shared among thermophiles.

**Table 2:** Statistical analyses of shared genes split into COGs. COG summary between bacteria where the number of reciprocal best hits has been corrected for genome size. Oxygen and temperature were assessed in a two way ANOSIM. GC content was assessed taking oxygen into account in a two way ANOSIM. For the ANOSIMs, a Bonferroni correction was used by dividing the *p* value needed (0.05) by 15 tests. Tests were only considered significant when *p* < 0.003. For Amino Acid Synthesis, all three variables (oxygen, temperature and %GC) were significant. Temperature and oxygen utilization were still signification after both genome size and %GC were corrected for simultaneously.

COG	O_2_ Utilization anaerobe vs aerobe	Temperature hyperthermo vs mesophile	GC Low vs high
Replication	*R* = 0.221 *p* = 0.001	*R* = 0.763 *p* = 0.001	NS
Transcription	*R* = 0.271 *p* = 0.001	NS	NS
Translation	*R* = 0.117 *p* = 0.002	*R* = 0.556 *p* = 0.001	NS
Amino Acid	*R* = 0.170 *p* = 0.001	*R* = 0.536 *p* = 0.001	*R* = 0.379 *p* = 0.001
Carbohydrate	NS	*R* = 0.655 *p* = 0.001	NS
Secondary metabolites	*R* = 0.451 *p* = 0.001	*R* = 0.166 *p* = 0.001	NS
Coenzyme	*R* = 0.253 *p* = 0.001	NS	NS
Energy	*R* = 0.571 *p* = 0.001	NS	*R* = 0.247 *p* = 0.001
Inorganic ion	*R* = 0.215 *p* = 0.001	*R* = 0.465 *p* = 0.001	NS
Lipid	NS	NS	NS
Nucleotide	NS	NS	*R* = 0.612 *p* = 0.001
Posttranslational modification	*R* = 0.402 *p* = 0.001	NS	NS
Cell wall	NS	NS	NS
Function unknown	*R* = 0.352 *p* = 0.001	*R* = 0.609 *p* = 0.001	NS
General function	*R* = 0.380 *p* = 0.001	*R* = 0.666 *p* = 0.001	NS

## Conclusions

We have identified bacteria with unusually large numbers of shared genes with archaea after accounting for the effect that genome size has on the number of shared genes (Fig. 5A). In general, these unusually large numbers of shared genes can be explained by horizontal gene transfer from archaea to bacteria (Figs. 4 and 7). More specifically, (hyper)thermophilic and anaerobic archaea are most likely to transfer genes to bacteria that live in high-temperature and anoxic habitats respectively (Fig. 7), though the genes transferred between anoxic and high temperature archaea and bacteria do differ (Table 2). In contrast, aerobic mesophiles appear to engage in very little inter-domain horizontal gene transfer. The most likely explanation for these results is that the (hyper)thermophilic and anaerobic organisms whose genomes were included in this study were isolated from environments (e.g., hot springs, oil wells, sediments) with high population densities where cells are in close proximity and therefore more likely to exchange genes. This study suggests that bacteria inhabiting bodies of water are less likely to contain genes transferred from archaea and that rates of horizontal gene transfer would be greatly underestimated if based only on aquatic organisms.

Furthermore, we have demonstrated the importance of adjusting for genome size when investigating other factors associated with the number of genes shared among genomes. Due to this genome size adjustment, this study is the first to be able to examine the effect of environment on horizontal gene transfer between archaea and bacteria on a broad scale.

##  Supplemental Information

10.7717/peerj.3865/supp-1 Supplemental Information 1 Supplemental Tables 1-5 of data used in analyses

10.7717/peerj.3865/supp-2 Supplemental Information 2 Residuals from the upper 95% CI for shared genes split into COGs

10.7717/peerj.3865/supp-3 Supplemental Information 3 Supplemental Figures 1–7
