# Supplementary material for: Effect of the environment on horizontal gene transfer between bacteria and archaea
Source: PeerJ. 2017 Sep 29;5:e3865. doi: 10.7717/peerj.3865 (PMC5624296; doi:10.7717/peerj.3865)
Supplement: Supplemental Information 3 [file peerj-05-3865-s003.pdf]

Supplemental Figures for: Effect of the environment on horizontal gene transfer between bacteria and archaea by C. A. Fuchsman, R.E. Collins, G. Rocap and W.J. Brazelton

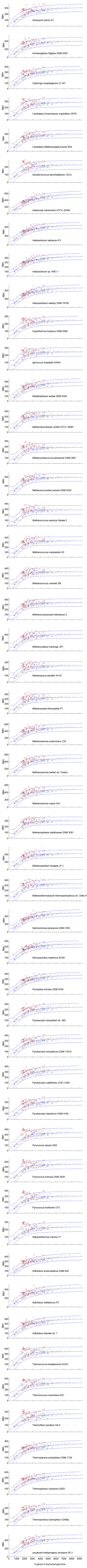
RBH
Aeropyrum pernix K1
Archaeoglobus fulgidus DSM 4304
Caldivirga maquilingensis IC-167
Candidatus Korarchaeum cryptofilum OPF8
Candidatus Methanoregula boonei 6A8
Desulfurococcus kamchatkensis 1221n
Haloarcula marismortui ATCC 43049
Halobacterium salinarum R1
Halobacterium sp. NRC-1
Haloquadratum walsbyi DSM 16790
Hyperthermus butylicus DSM 5456
Ignicoccus hospitalis KIN4/I
Metallosphaera sedula DSM 5348
Methanobrevibacter smithii ATCC 35061
Methanocaldococcus jannaschii DSM 2661
Methanococcoides burtonii DSM 6242
Methanococcus aeolicus Nankai-3
Methanococcus maripaludis S2
Methanococcus vannielii SB
Methanocorpusculum labreanum Z
Methanoculleus marisnigri JR1
Methanopyrus kandleri AV19
Methanosaeta thermophila PT
Methanosarcina acetivorans C2A
Methanosarcina barkeri str. Fusaro
Methanosarcina mazei Go1
Methanosphaera stadtmanae DSM 3091
Methanospirillum hungatei JF-1
Methanothermobacter thermautotrophicus str. Delta H
Natronomonas pharaonis DSM 2160
Nitrosopumilus maritimus SCM1
Picrophilus torridus DSM 9790
Pyrobaculum aerophilum str. IM2
Pyrobaculum arsenaticum DSM 13514
Pyrobaculum calidifontis JCM 11548
Pyrobaculum islandicum DSM 4184
Pyrococcus abyssi GE5
Pyrococcus furiosus DSM 3638
Pyrococcus horikoshii OT3
Staphylothermus marinus F1
Sulfolobus acidocaldarius DSM 639
Sulfolobus solfataricus P2
Sulfolobus tokodaii str. 7
Thermococcus kodakarensis KOD1
Thermococcus onnurineus NA1
Thermofilum pendens Hrk 5
Thermoplasma acidophilum DSM 1728
Thermoplasma volcanium GSS1
Thermoproteus neutrophilus V24Sta
uncultured methanogenic archaeon RC-I
0 1000 2000 3000 4000 5000 6000 7000 8000 9000
genes in bacterial genome

Figure S1. The number of shared genes between archaea and bacteria and bacterial genome size were fitted using non-linear regression (center blue line) and 2 standard deviations from the mean are also shown to visualize the range (upper and lower blue lines). Color scheme of the points reflect growth temperature: thermophiles are red, mesophiles are grey and psychrophiles are blue. The name of the relevant archaea appears on the plot.

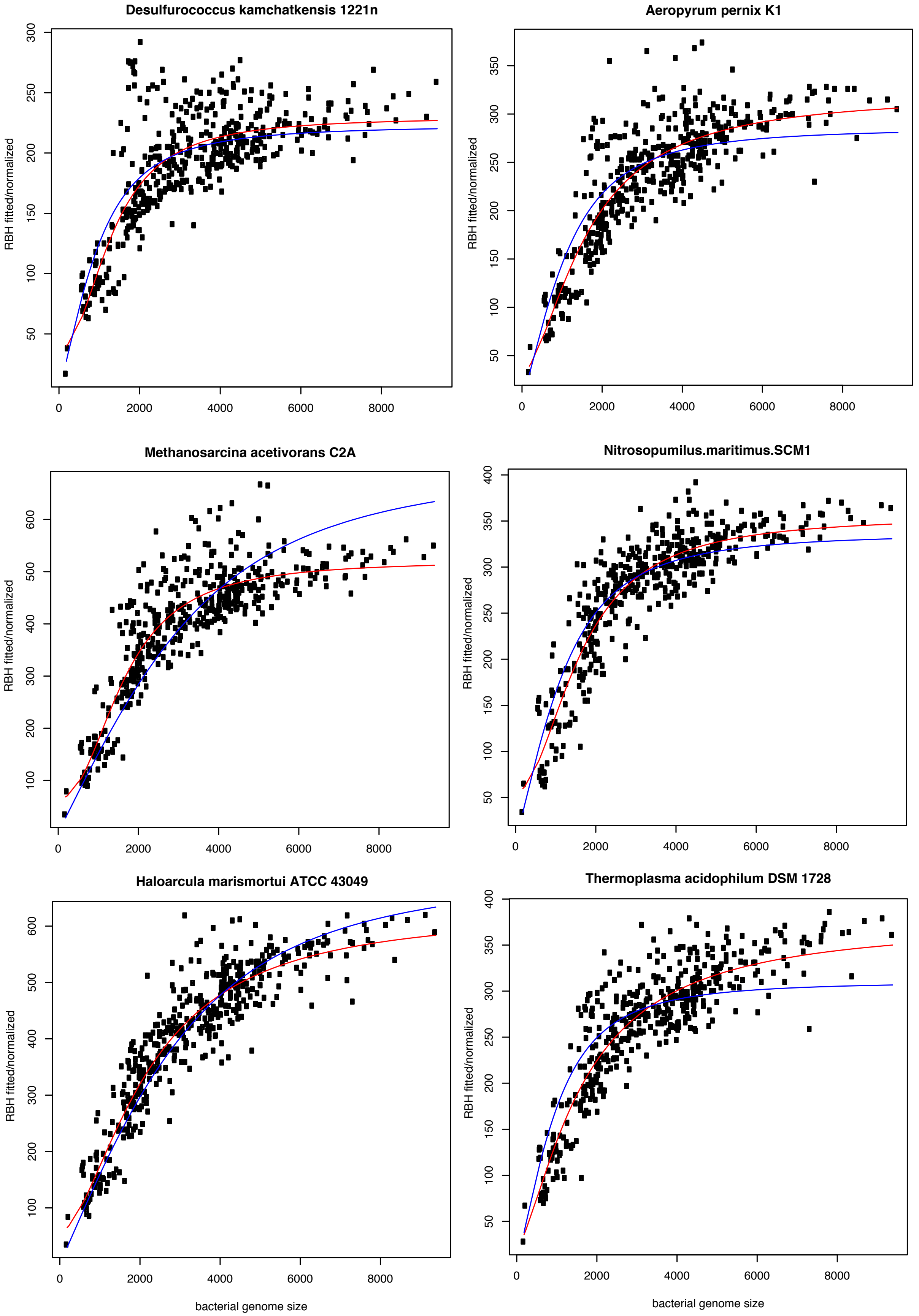

Desulfurococcus kamchatkensis 1221n
Aeropyrum pernix K1
Methanosarcina acetivorans C2A
Nitrosopumilus.maritimus.SCM1
Haloarcula marismortui ATCC 43049
Thermoplasma acidophilum DSM 1728
RBH fitted/normalized
bacterial genome size
0
2000
4000
6000
8000
50
100
150
200
250
300
350
400
500
600

Figure S2. A comparison of fits for select archaeal genomes. The Korbel normalization (blue line) is weighed average =sqrt(2)*A*B /(A^2+B^2) where A and B are the genome size of the archaeal and bacterial genomes (Korbel et al 2002). The four-parameter log-logistic function (red line) was used in further analyses.

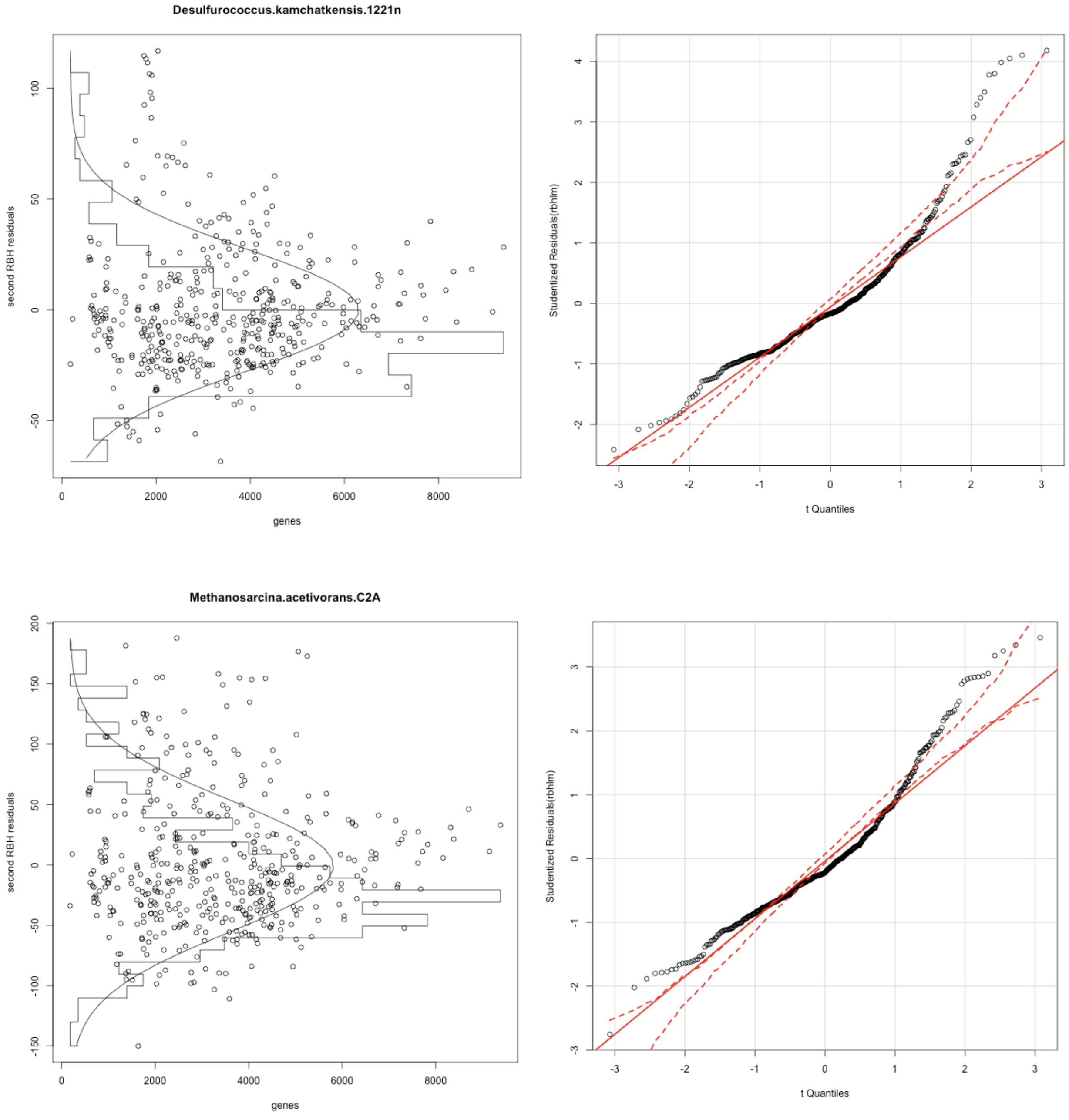

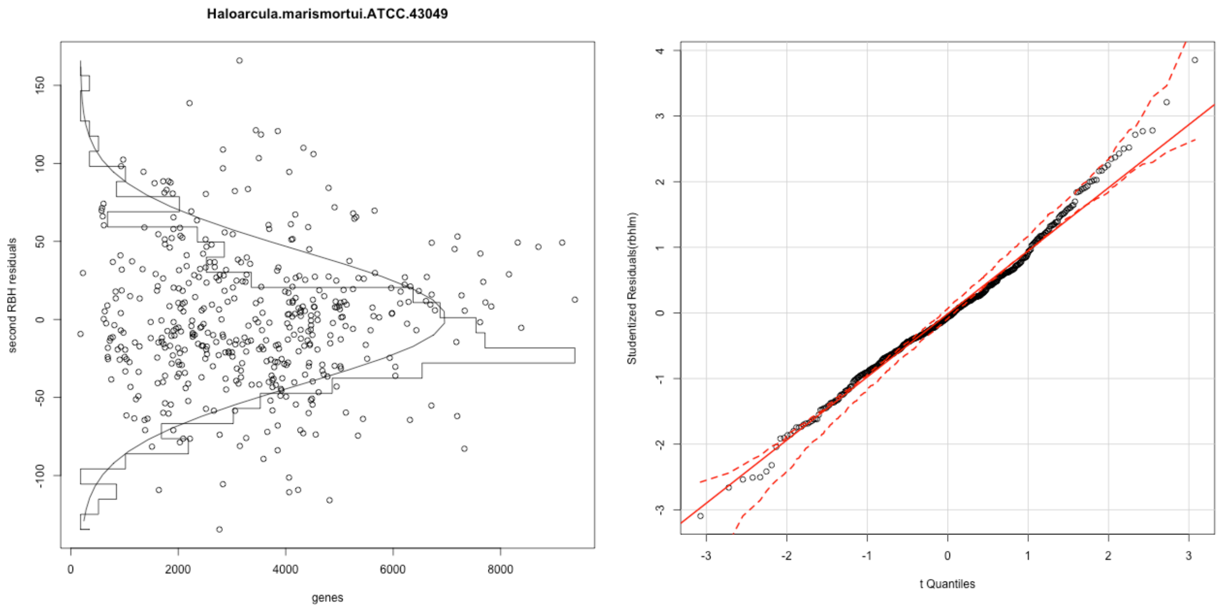

Figure S3. Plots indicating that the distribution of RBH residuals in not normal. The non-normality of the residuals serves as a test of our claim that some bacteria have non-random contributions of archaeal genes from horizontal gene transfer. When more outliers with high RBH are present (as in the case of *Desulfurococcus kamchatkensis*), the distribution of the residuals is skewed, and the QQ plot shows a marked deviation from linearity. In contrast, *Haloarcula marismortui*, which we predict to have had low rates of HGT with bacteria, shows a nearly linear QQ plot and highly normal distribution of RBH with genome size.

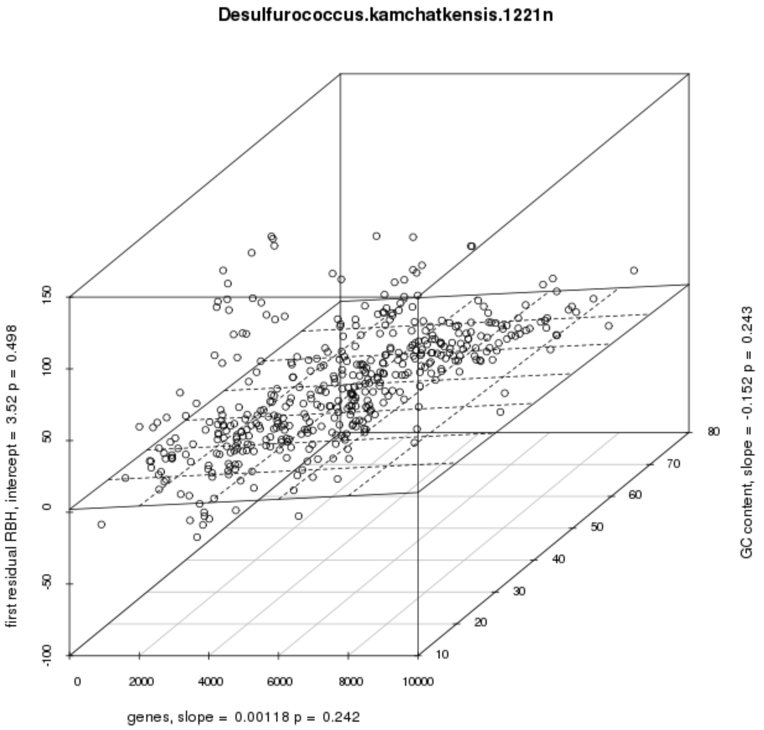
Desulfurococcus.kamchatkensis.1221n
first residual RBH, intercept = 3.52 p = 0.498
GC content, slope = -0.152 p = 0.243
genes, slope = 0.00118 p = 0.242

B.

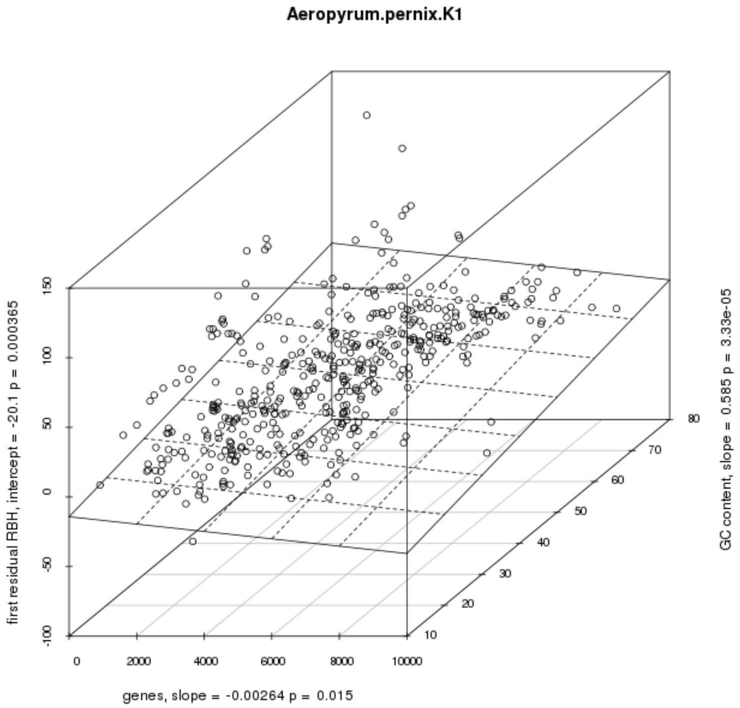
Aeropyrum.pernix.K1
first residual RBH, intercept = -20.1 p = 0.000365
GC content, slope = 0.585 p = 3.33e-05
genes, slope = -0.00264 p = 0.015

C.

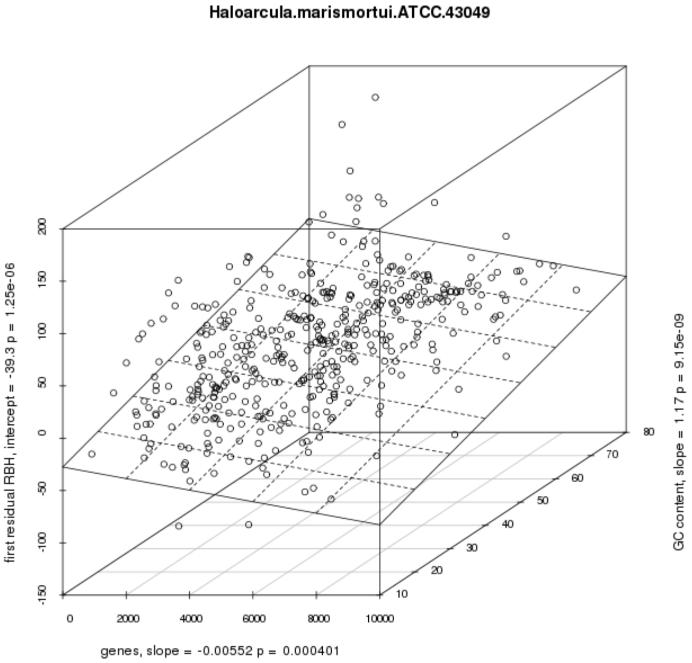

Figure S4. The affect of GC was removed from the residuals for each archaea by fitting a plane to the three axes: number of genes in the bacterial genome, reciprocal BLAST hits, and GC content. Examples include A) anaerobic hyperthermophile *D. kamchatkensis* B) aerobic hyperthermophile *A. pernix* and C) Extremely halophilic aerobic mesophile *H. marismortui*.

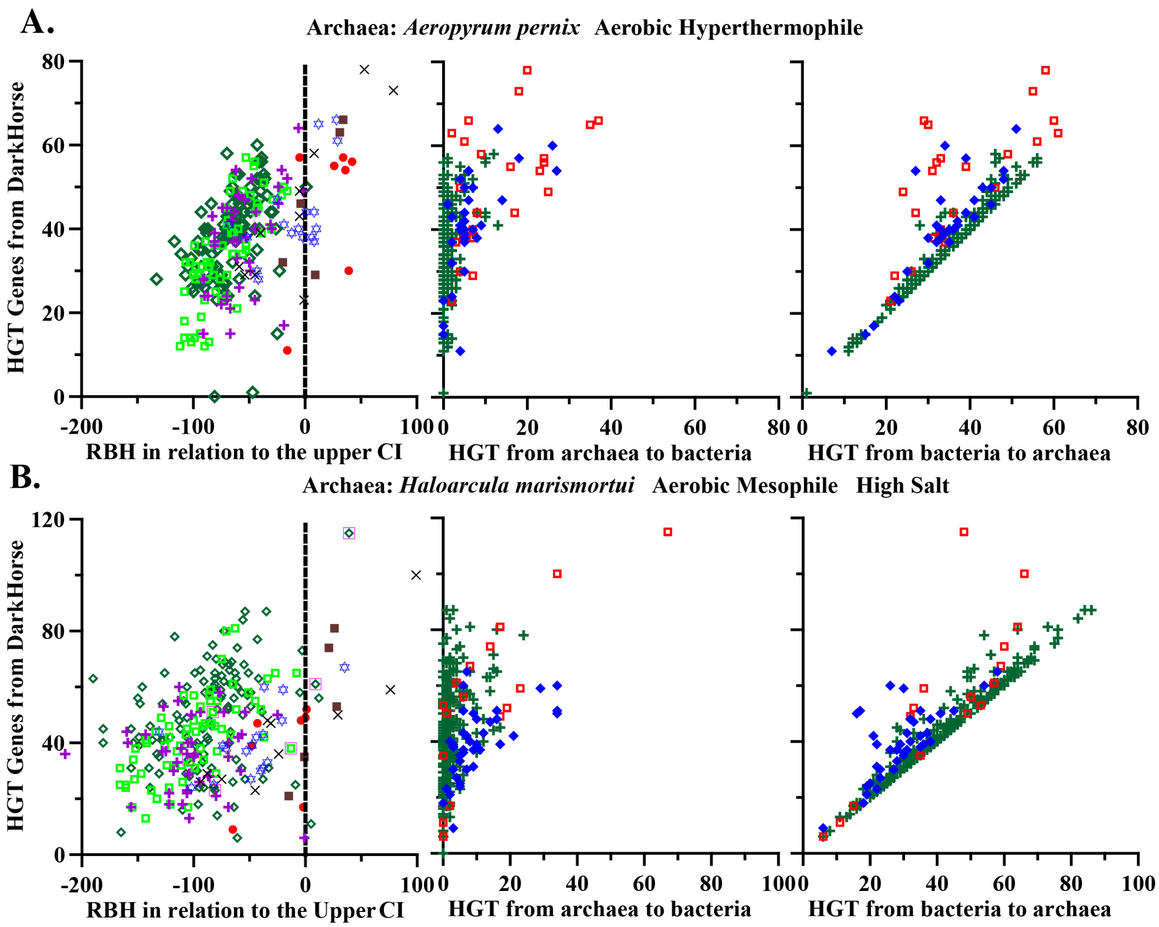

Figure S5. Examples of HGT for aerobic hyperthermophile *Aeropyrum pernix* (A) and the aerobic mesophile but extremely halophilic archaea *Haloarcula marismortui* (B). In the first column the number of potentially horizontally transferred genes as determined from the DarkHorse algorithm is compared to the number of reciprocal best hits between bacteria and each archaeon. A positive value on the x-axis indicates the RBH count was greater than 2 standard deviations from the mean. Color coding describes the lifestyle of the bacteria. A pink box in B indicates extremely halophilic bacteria. In the second column, the number of potentially transferred genes is compared to the number of those genes transferred from archaea to bacteria. In the third column, the number of potentially transferred genes is compared to the number of those genes transferred from bacteria to archaea. Color-coding describes whether the bacterium forms a pair with the archaeon listed (red), with any archaea in this study (blue), or not with any archaea (green).

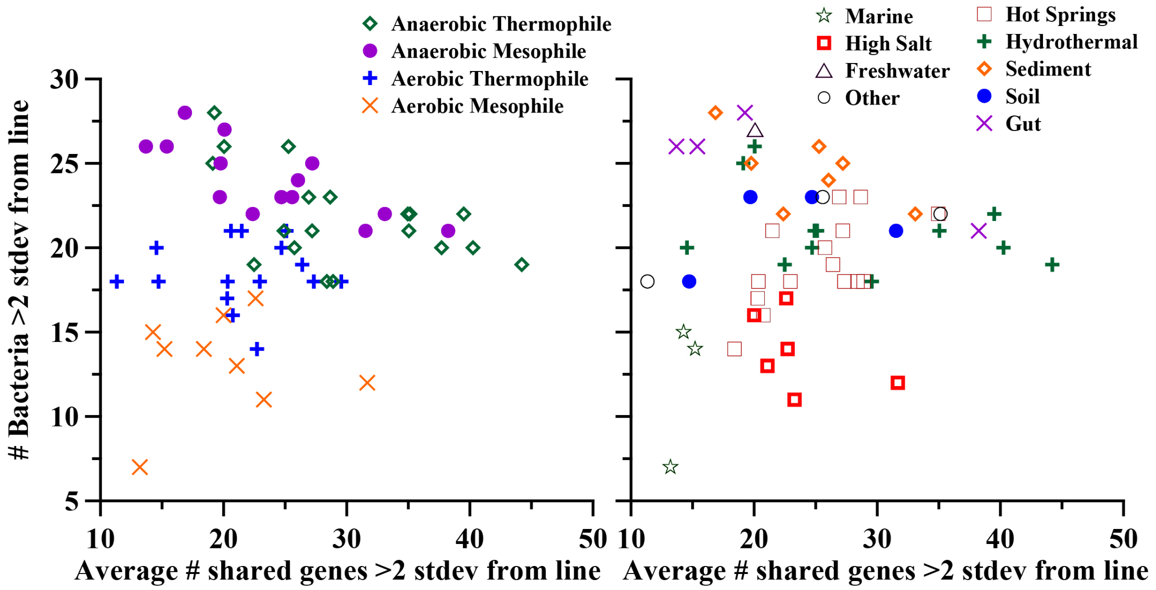

Figure S6. The number of archaea-bacteria pairs enriched in shared genes (RBHs) and the average number of reciprocal best hits in each pair, plotted by archaeon. In A) color coding describes the phenotypes of the archaea. In B) color coding describes the environments the archaea were isolated from.

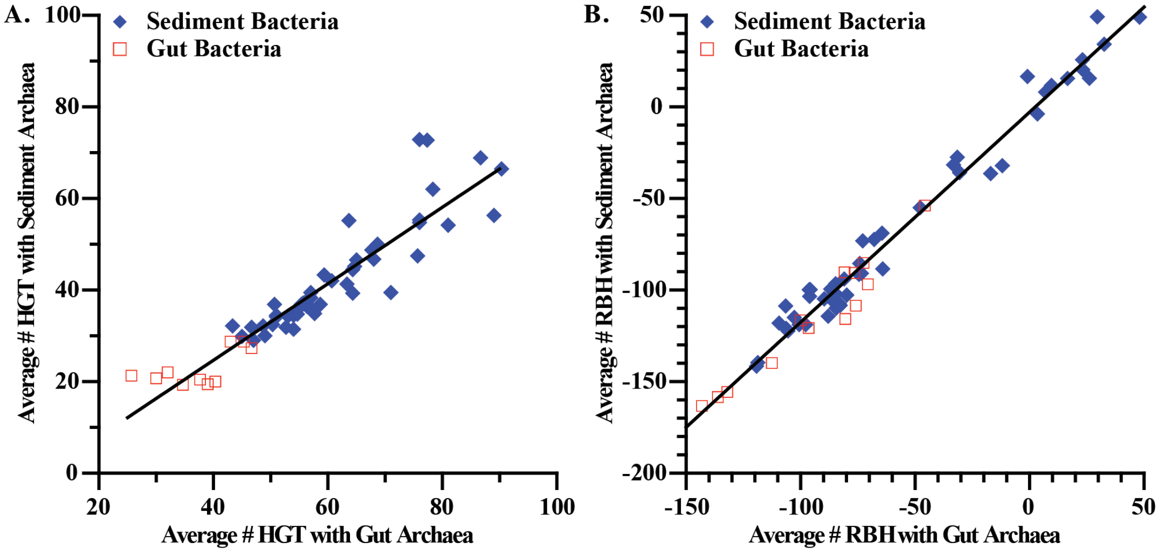

Figure S7. Comparison of transferred (A.) and shared (B.) genes with habitat. Particularly, we compare genes in sediment and gut bacteria and sediment and gut archaea. HGT stands for horizontally transferred genes, and RBH stands for reciprocal best hits or shared genes.
